# Clival chordoma in a young male patient: a case report

**DOI:** 10.11604/pamj.2020.37.59.24836

**Published:** 2020-09-15

**Authors:** Aref Zribi, Sonia Ben Nasr, Aya Khemir, Faten Gargouri, Ichrak Ben Abdallah, Issam Msakni, Sana Fendri, Mehdi Balti, Basma Laabidi, Abderrazek Haddaoui

**Affiliations:** 1 Department of Medical Oncology, Faculty of Medicine of Tunis, University of Tunis El Manar, Military Hospital of Tunis, Montfleury, Tunis, Tunisia,; 2 Department of Pathology, Faculty of Medicine of Tunis, University of Tunis El Manar, Military Hospital of Tunis, Montfleury, Tunis, Tunisia.

**Keywords:** Chordoma, clival, treatment, radiotherapy, surgery

## Abstract

Chordoma is a rare malignant tumor of the spine. We report the case of a 26-year-old man who presented with facial paralysis and upper limbs paresthesia. Cerebral CT-scan and cerebro-spinal MRI revealed a 58mm locally advanced middle clival mass with deviation of median cerebral structures. Endoscopic biopsy concluded to a chondroid chordoma. Skeletal survey and thoraco-abdomino-pelvic CT-scan were normal. Treatment consisted in complete surgical removal of the tumor followed by adjuvant radiotherapy. The patient is alive free of disease with a follow up of 12 months.

## Introduction

Chordoma is a slowly evolving and locally aggressive rare cancer. It accounts for four percent of primary bone cancers. It arises from remnants of the notochord and may occur anywhere in the axial skeleton with predilection for the sacrum (65% of cases). Chordomas are rarely diagnosed before the age of 40 [1]. Clival chordomas represent 35 to 40% of cases and are difficult to treat because of the complexity of neighboring anatomical structures, local aggressivity and high recurrence rates [2]. Three histological subtypes are described: classical, chondroid and dedifferentiated. The chondroid subtype has a better prognosis [1]. Clival chordomas´ management is not yet consensual. The role of adjuvant chemotherapy and radiotherapy in improving disease free survival was reported [1,2]. We report a new observation of clival chordoma in a 26-year-old patient.

## Patient and observation

**Figure 1:**
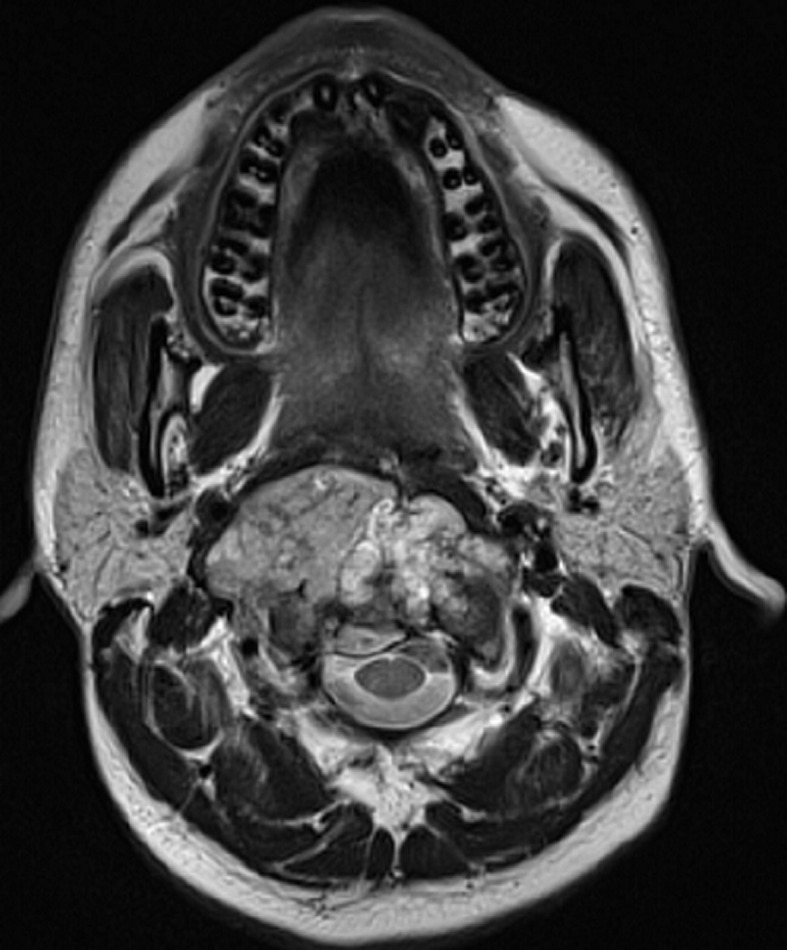
cerebro-spinal MRI, a locally aggressive lesion of the clivus invading the right cavernous sinus (axial cut of brain)

**Figure 2:**
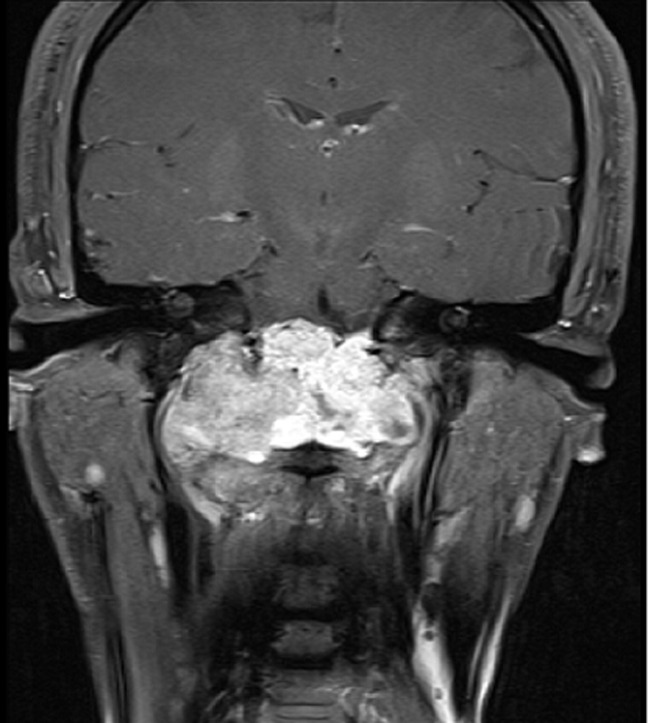
cerebro-spinal MRI, a locally aggressive lesion of the clivus invading the right cavernous sinus (coronal cut of brain)

**Figure 3:**
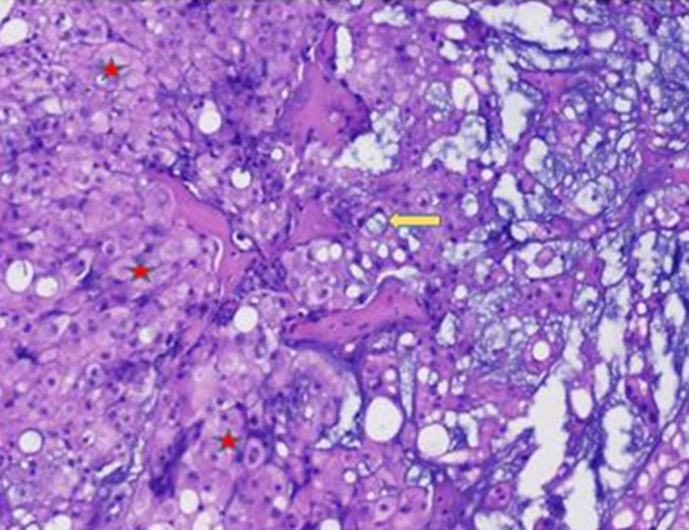
(HEx100) chondroid chordoma the tumor cells have abundant eosinophilic cytoplasm and prominent cytoplasmic borders (star). The characteristic physaliferous cells have vacuolated cytoplasm (arrow)

**Figure 4:**
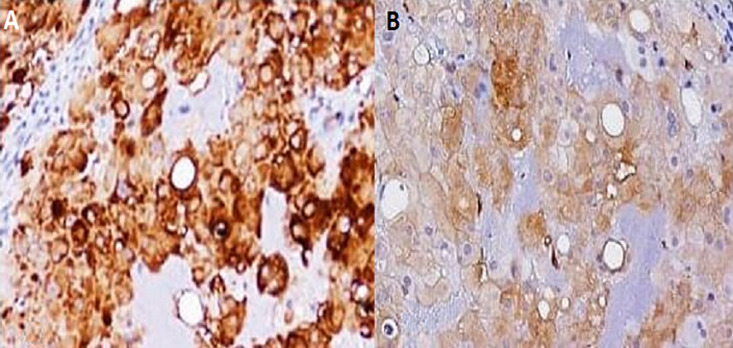
(A) immunohistochemical staining of tumour tissue, the tumour cells showed a strong positive reaction for the cytokeratin; (B) S-100-antibody

A 26-year-old man with no medical history presented with orofacial paralysis and upper limbs paresthesia. Cerebral CT-scan revealed a locally advanced 58mm osteolytic tumor in the middle of the clivus. The tumor invaded the oropharynx, the para-pharyngeal spaces and the spinal canal with brain stem compression. Cerebro-spinal MRI showed a locally aggressive lesion of the clivus invading the right cavernous sinus, T1 isointense and T2 hyper intense with heterogenous enhancement on post-contrast images (Figure 1 and Figure 2). Pathology report based on conventional hematoxylin and eosin staining showed a characteristic epithelioid and partially physaliferous tumor cells arranged in lobules. Lobules were separated by thin fibrous septae embedded within a myxoid matrix resembling neo-plastic hyaline cartilage (Figure 3). Immunohistochemical staining showed strong positivity for pancytokeratin. Tumor cells showed also a weak cytoplasmic and nuclear positivity for S100 (Figure 4). Therefore the diagnosis of chondroid chordoma of the clivus was retained by pathologists. Staging workup including bone scan and thoraco-abdomino-pelvic CT-scan did not reveal any metastases. Surgical resection of the tumor followed by radiotherapy was decided in a multidisciplinary concertation meeting. A subtotal resection of the tumor mass was made via a retrosigmoid craniotomy approach. Pathology report confirmed the diagnosis of chondroid chordoma. The postoperative course was uneventful. The patient reported a progressive improvement of initially reported symptoms. Four months later, the patient received γ-knife radiation therapy with good tolerance. Seventh nerve palsy recovered completely. The patient is alive free of disease after a 12-month follow-up.

## Discussion

Chordoma is a rare bone cancer of embryologic origin. Its incidence does not exceed 0.08 per 100.000. It occurs rarely before the age of 40 [1]. The mean age is 47.7 years. Patients present usually with headaches, cranial neuropathy and diplopia. Epistaxis, subarachnoid hemorrhage and cerebrospinal fluid rhinorrhea are less frequently reported [2]. Tumor staging is based on CT-scan and MRI. Chordomas are usually well-circumscribed hypoattenuating masses, expanding to adjacent soft-tissues with osteolytic bone lesions on CT-scan. Intra-tumoral calcifications may be seen and are characteristic of the chondroid variant. MRI is superior to CT-scan in evaluating tumor extension to adjacent soft tissues. However, MRI does not describe well cortical bone involvement and/or destruction. Yet, it remains the best imaging technique in evaluating tumor size and extension after radiation therapy [3]. Pathologic examination of biopsies specimens or exised tumor masses is curcial to identify the tumor nature and judge the completeness of surgical excision. Tumor cells are arranged in sheets, cords or float singly within an abundant myxoid stroma in the classic variant of chordoma. The stroma may contain chondroid areas reminiscent of hyaline cartilage in the chondroid variant [4]. Tumor cells have typically an abundant pale vacuolated cytoplasm (the classic “physaliferous cells”). Nuclei show mild to moderate atypia.

Mitoses are infrequent. Dedifferentiated chordoma, the third histologic variant of chordomas, is defined by the presence of areas of high grade sarcoma. It accounts for less than 5% of all chordomas. Immunohistochemical staining shows positivity of tumors cells for S100 protein, pan-keratin, low molecular cytokeratins and Epithelial Membrane Antigen (EMA) [4]. In Bai and co-authors´ study [5] patients were divided into two groups based on the electron micrograph examination. The first group was designed as «cell dense type chordoma» and the second group as «matrix rich cell type chordoma»; the cell dense type chrodoma had higher mortality rate than matrix rich chordomas. In this study authors concluded that cell dense type is associated with more aggressive behavior, proliferating potential, higher risk of recurrence and shorter survival. Metastases were reported in 3 to 48% of cases in literature. They were mostly associated with sacrococcygeal (45%) rather than clival chordomas. The two most common metastatic sites are lungs and lymph nodes followed by liver and bone. Cardiac metastases have also been reported [6]. Management of clival chordomas is not well codified because of the rarity of the tumor. Hence, cases must be discussed in multidisciplinary concertation meetings including medical and radiation oncologists, pathologists and surgeons. Tumor locoregional extension is an important factor to state on the necessity of neoadjuvant chemotherapy and the possibility of complete surgical excision [7]. Craniotomy with extensive surgical resection has improved patients´ prognoses at the cost of severe neurological damages [2].

Recently, endoscopic endonasal resection has been suggested as a less invasive approach. Yet, given the rarity of clival chordomas, we´re lacking data comparing both approaches [2]. Kim *et al*. evaluated the endoscopic endonasal approach in the treatment of basal skull malignant tumors in a group of 37 patients. The authors concluded that endoscopic endonasal approach is associated with better surgical results and clinical outcome. However, this technique could not be applied to patients with paramedian clival chordomas because of the difficult accessibility to this area [6]. Shimony *et al*. [8] compared the two surgical approches in the treatment of skull base chordomas. They noted the presence of residual tumor in all craniotomy cases. They also concluded that endoscopic endonasal resection is the best approach for chordomas located in the midline without lateral extension. In some cases, different surgical approaches may be associated to ensure optimal surgical resection [8]. Multidisciplinary concertation meetings are crucial for clival chordomas´ management. Freeman *et al*. [9], compared therapeutic results of clival chordomas in two groups of patients. In the first group patients were treated in multidisciplinary centers. In the second one patients were treated outside multidisciplinary centers. They concluded to an increased risk of recurrence and disease progression in the second group of patients. Liang *et al*. identified FGFR1 overexpression and CDK4 and ERBB3 copy-number duplication as genetic alterations inducing tumor proliferation [10]. Efficacy of ponatinib was reported in patients with FGFR1 overexpression. Pazopanib and palbociclib exhibited a higher efficiency in cases of CDK4 duplication while gefitinib was more efficient in cases of ERBB3 duplication [10].

## Conclusion

Clival chordomas are rare slow-growing tumors exhibiting a locally aggressive behavior and high rates of local recurrences. Their management remains to be clarified and should be based on a multidisciplinary team approach. Whenever possible, complete surgical removal of the tumor mass should be performed to ensure better local control and long-term survival.
